# Autistic traits, sensory sensitivity and eating disturbances in a sample of young adults referring to a generalized mental health clinic

**DOI:** 10.1007/s40519-024-01639-7

**Published:** 2024-01-23

**Authors:** Veronica Nisticò, Gianmarco Ingrosso, Francesco Lombardi, Elia Chiudinelli, Giulia Bianchini, Raffaella Faggioli, Angelo Bertani, Orsola Gambini, Benedetta Demartini

**Affiliations:** 1 Dipartimento di Scienze della Salute, Università degli Studi di Milano, Presidio San Paolo, via A. Di Rudinì, 8, 20142 Milano, Italy; 2 “Aldo Ravelli” Research Center for Neurotechnology and Experimental Brain Therapeutics, University of Milan, Milano, Italy; 3 Dipartimento di Psicologia, Università degli Studi di Milano-Bicocca, Milano, Italy; 4 Unità di Psichiatria 51 e 52, Presidio San Paolo, ASST Santi Paolo e Carlo, Milan, Italy; 5 Dipartimento Salute Mentale e Dipendenze, Centro Giovani “Ettore Ponti”, ASST Santi Paolo e Carlo, Milan, Italy

**Keywords:** Eating disorders, Autistic traits, Sensory sensitivity, Young adults

## Abstract

**Purpose:**

The relationship between autistic traits and eating disturbances has been given considerable attention over the last decades. The rise of a dimensional approach to psychopathology has expanded the way we think about autism, acknowledging that subthreshold autistic manifestations span across the general population and are more pronounced in psychiatric patients. Here we investigated the prevalence of eating disorders and its potential relationship with autistic traits and sensory sensitivity in a group of patients who were referred for the first time to a mental health outpatient clinic, without a formal diagnosis yet.

**Methods:**

259 young adults (between 18 and 24 years old) completed: the Eating Attitude Test (EAT-26), the Swedish Eating Assessment for Autism Spectrum Disorders (SWEAA), the Autism Quotient (AQ), the Ritvo Autism Asperger Diagnostic Scale-Revised (RAADS-R), and the Sensory Perception Quotient—Short Form 35 item (SPQ-SF35).

**Results:**

23.55% of participants scored above the cut-off at the EAT-26, suggesting that they presented a risk for eating disorders and should be assessed by a specialized clinician; associations emerged between hypersensitivity in the touch and vision domain and both the EAT-26 and the SWEAA; the presence of autistic traits was largely associated with eating disturbances.

**Conclusions:**

This study underlines the significance of the eating domain as a central psychopathological feature in the distress experienced by young adults with general psychiatric symptoms and psychological suffering; it adds evidence to the association between autistic traits and eating disorders and opens to new research questions about the role of subthreshold autistic traits in general psychopathology.

*Level of evidence:* Level I: Evidence obtained from experimental studies.

**Supplementary Information:**

The online version contains supplementary material available at 10.1007/s40519-024-01639-7.

## Introduction

The relationship between autistic traits and eating disturbances is still unclear, although the matter has been given considerable attention over the last decades. Autism Spectrum Disorders (ASD) are defined as a group of neurodevelopmental conditions characterized by difficulties in social interaction and communication, restricted and repetitive patterns of behaviours and interests, and altered sensory sensitivity, whereas Eating Disorders (ED) are characterized by abnormal eating habits and attitudes towards food and body image, that significantly impact on individual’s physical and psychological well-being [1].

The interest in the association between ED and ASD has been sparked by several factors, such as the identification of a familiar aggregation of these conditions [2], the frequently reported evidence of autistic traits among patients with ED, and of abnormal eating behaviours in individuals with ASD [3–5]. The two conditions also share common psychopathological features, such as cognitive rigidity, atypical social cognition, and difficulties in emotion processing [6]. Moreover, the existence of alterations in sensory sensitivity, a typical feature observed both in patients with ASD and in patients with ED [7], has been linked with higher ED severity, difficulties in regulating emotions, and distorted perception of body image; notably, it often remains present despite treatment and weight restoration [8, 9]. Though both full-blown ASD forms and subthreshold autistic traits have been identified in ED beyond Anorexia Nervosa (AN) [5], most research has concentrated on the latter. Notably, studies revealed that individuals with AN score similarly to those with ASD on the Autism Spectrum Quotient (AQ), a validated self-report measure of autistic traits [4, 10]. During acute AN phase, starvation and underweight may heighten pre-existing autistic traits, framing the ASD-AN association as a combination of trait and state [11]. Conversely, individuals recovered from AN often continue to manifest ASD-related symptoms compared to healthy controls [12]. Hence, while autistic traits may vary in severity during the clinical course of AN [13], they seem to maintain a relatively consistent pattern over time and are not evidently linked to body weight and potential recovery [14].

Regarding sensory sensitivity alterations, while there is a wealth of scientific research in the ASD field, the body of literature addressing sensory sensitivity in ED is comparatively limited. Individuals with ASD often exhibit anomalies in sensory reception, including hyper- and hypo-responsiveness, with approximately 90% experiencing atypical sensory responses [1, 15–17]. Such perceptual anomalies often predict the severity of autistic traits, ultimately impacting on social interactions [18]; common behaviours that can be imputed to hypersensitivity are: scarcely maintaining eye contact, focusing on the objects’ details, covering one’s own ears for everyday sounds, being visibly bothered by certain smells, and refusing to eat certain food or to wear specific fabrics [17]. Scientific evidence illustrates sensory anomalies across the five senses domains. In the visual domain, ASD individuals detect single details faster and prefer pixel-level saliency in images [19, 20]. Tactile sensitivities include heightened response to vibrations and thermal pain [21]. Auditory anomalies involve an increase in the incidence of individuals with absolute pitch [22]. Olfactory challenges result in lower accuracy in recognizing odours [23]. Contradictory findings emerge in taste perception studies, with reports of reduced accuracy in identifying bitter, sweet, and sour tastes [16, 24]; such sensory difficulties contribute to reported food selectivity, particularly regarding textures in children with ASD [25, 26]. With respect to ED, most of the studies have proven a range of differences in both overall sensory sensitivity and specific sensory domains, with many of these alterations tending to persist despite treatment [27]. Furthermore, it has been hypothesized that the core symptoms of ED could be linked to a deficiency in multisensory processing [28], and that in patients with AN sensory hypersensitivity may contribute to the distorted Body Image that these patients perceive. In turn, this can suggest that altered sensitivity may potentially serve as a risk factor for the development of ED [29].

The present study builds upon a series of previous findings by our research group. First, we found that adults with ASD without intellectual disability, compared to neurotypical healthy controls, showed not only a higher prevalence of autistic-like eating disturbances (as evaluated by the Swedish Eating Assessment for Autism Spectrum Disorders, SWEAA, [30]), but also of other ED symptoms and concerns, assessed with the Eating Attitude Test (EAT-26) [31]. Consistently with these findings, it has been found that participants with ASD without intellectual disabilities, particularly women, experienced more eating disturbances than neurotypical controls, as per the SWEAA scale [32]. Hence, we conducted a study directly comparing women with ASD without intellectual disability and patients with ED; our findings indicated that while the ASD group did not exhibit the same level of ED symptoms, as per EAT-26, as diagnosed ED patients, both groups reached comparable scores on the SWEAA, indicating shared alimentary challenges associated with autism. Notably, in both groups these scores were higher compared to those of neurotypical controls [33]. Considering the aforementioned presence of altered sensory sensitivity in both ASD and ED, and given that the association between sensory abnormalities and eating behaviors has primarily been explored in children diagnosed with ASD [25], but not in adults, we investigated this relationship in a group of adult individuals with a diagnosis of ASD [34], and in a group of women diagnosed with different ED (including AN, BN, BED, UFED) [35]. In the ASD group, sensory sensitivity alterations were associated both with ED symptomatology, as per EAT-26, and with autistic-like eating behaviours, as per SWEAA; in particular, hypersensitivity in the vision domain was linked to higher levels of both ED symptoms and autistic-like eating behaviours, while hyposensitivity in the taste domain was associated with higher levels of ED symptoms only [34]. In the ED group, we found a 12% prevalence of autistic traits (considered present when scoring above the cut-off on both the Autism Quotient (AQ) and the Ritvo Autism Asperger Diagnostic Scale-Revised (RAADS-R)); moreover, the scores of the RAADS-R substantially contributed to explain the relationship between the sensory thresholds in the 5 domains (measured via the SPQ-SF35 questionnaire) and both autistic-like eating behaviours and ED symptoms severity. Of note, in this case, hypersensitivity in the taste domain was associated with both ED severity and autistic-like eating behaviours, while hypersensitivity in the vision domain was associated with autistic-like eating behaviours [35].

Although preliminary, these findings have prompted new research inquiries about the role of autistic traits in the relationship between altered sensory sensitivity and dysfunctional eating behaviours, as well as with other psychiatric symptomatology. As a matter of fact, the rise of a dimensional approach to psychopathology has expanded the way we think about ASD, acknowledging that subthreshold autistic manifestations span across the general population, and are more pronounced in clinical groups of patients with several psychiatric conditions [36].

Considering this background, the aim of this study was to investigate the prevalence of ED symptomatology and its potential relationship with autistic traits and sensory sensitivity in a group of patients who were referred for the first time to a mental health outpatient clinic, seeking help for unspecified psychiatric and psychological suffering, in the very first phases of patients’ care, when a proper diagnosis had not been reached yet.

## Materials and methods

### Participants

The study population comprised all the adolescent and young-adult patients referring to the “Centro Giovani Ettore Ponti” (ASST Santi Paolo e Carlo, Milan, Italy), a specialized consultative clinic for individuals aged between 18 and 24 years old presenting psychiatric symptoms, beginning from the 24th September 2021 until the 30th September 2022. Exclusion criteria were: (i) age less than 18 years old; (ii) inability to understand the instruction of the task. At the end of the recruitment phase, a total of 259 patients met the inclusion criteria; this group included both new patients and subjects already undergoing follow-up in the clinic. All participants gave their written informed consent for the study and were free to leave at any time without giving further explanation. The local Ethics Committee reviewed and approved the study protocol.

### Procedure

Due to the concomitant COVID-19 pandemic, data were collected through an online questionnaire. During the first in-person consultation, a psychiatrist, who was informed about the purpose of the study, asked the participants to sign the informed consent and gave them a link with a series of questionnaires to be completed within the following 48 h; participants were provided with the clinician's contact information for any questions potentially emerging during the questionnaire completion process. At the end of the first consultation, before receiving the results of the questionnaires, the clinician categorized their symptomatology according to the following categories: anxiety symptomatology; personality disorders symptomatology; potential mood disorders; potential disorders with onset during childhood or neurodevelopmental condition; psychotic symptoms; eating behavior related symptoms; multiple symptoms to be further investigated. After receiving the results of the questionnaire, diagnostic findings, as deemed significant by the clinician, were subsequently discussed in follow-up consultations.

The online questionnaire first included questions about sociodemographic information such as gender, height, weight, sexual orientation, education level, employment, and living environment. Subsequently, each participant completed the following four validated self-report questionnaires.

*The Autism Quotient (AQ).* It is a 50-item self-report questionnaire measuring the degree to which an adult without intellectual disabilities presents autistic traits; a Total Score of 32 or more is indicative of the presence of ASD traits [10].

*The Ritvo Autism Asperger Diagnostic Scale-Revised (RAADS-R).* It is an 80-item validated instrument designed to assist clinicians diagnosing ASD in adults; a Total Score of 66 or more suggests that the participants should be further assessed for ASD [37].

*The Eating Attitude Test-26 items (EAT-26)*. It is a validated standardized self-report measure of symptoms and concerns specific to eating disorders. It consists of 26 items, to be replied on a 6-point Likert Scale ranging from “Never” to “Always”. Except for item 26, whose scoring is reversed, answers “Never”, “Seldom” and “Sometimes.” Are scored 0, “Often” is scored 1, “Normally” is scored 2 and “Always” is scored 3. A Total Score and three subscales (Dieting, Bulimia, and Oral Control) were calculated. Participants scoring above 20 were considered at risk of presenting an Eating Disorder, and hence recommended a specific clinical evaluation with an expert clinician; the subscale Dieting evaluates the participant’s attention to calories ingested and burned doing physical exercise, their desire to be thin, and the sense of guilt after eating (e.g., “I feel extremely guilty after eating”); the subscale “Bulimia” assess the presence of bulimic and binge-eating symptoms and concern about food (e.g., “I have gone on eating binges where I feel that I may not be able to stop”); the subscale “Oral control” investigates the participants’ self-control over eating and the perceived pressure from others to gain weight (e.g. “I feel that other pressure me to eat”) [38, 39].

*The Swedish Eating Assessment for Autism Spectrum Disorders (SWEAA).* This is a 65-item self-report scale assessing the presence of autistic-like eating behaviours; participants are asked to reply to each item on a 5-point Likert Scale, ranging from 0 (Never) to 4 (Always). A Total Score and the following subscales were calculated: (i) Perception, assessing sensitivity to sensory input related to food, such as smell, taste, texture or sound (e.g. “I am oversensitive to certain flavours”; (ii) Motor Control, investigating issues in different aspects of movement that can influence eating behaviour, such as problems chewing or spilling, as well as table manners (e.g. “I spill while I eat”); (iii) Purchase of Food, assessing the grade of control over purchases, such as brands or type of groceries (e.g. “My food must be of a certain brand”); (iv) Eating Behaviour, assessing participants’ selectivity in eating, their limited repertoire and their difficulties in trying new foods (e.g. “I only eat a limited menu, maximum of 10 dishes”); (v) Mealtime Surrounding, investigating routines around mealtime such as where to eat and how cutlery is placed (e.g. “I find it difficult to change seats at the dining table); (vi) Social Situation at Mealtime, assessing difficulties in adapting their own behaviour to that of others or enjoying company during a meal (e.g. “I look down at my food most of the time during a meal”); (vii) Other Behaviour Associated with Disturbed Eating, evaluating the presence of other typical symptoms of an eating disorder (e.g. “I induce vomiting after meals”); (viii) Hunger/Satiety, assessing the ability to perceive when hungry or full (e.g. “I feel when I am hungry”) (ix) Simultaneous Capacity, evaluating the difficulties to do two things simultaneously during a meal (e.g. “I find it difficult to do two things simultaneously during a meal, like chewing and cutting the food); (x) Pica, investigating whether participants eat inedible things (e.g. “I eat things that others consider inedible, such as mortar or soil”), (xi) Autism Quotient, including items specifically selected by the SWEAA authors from the validated questionnaire Autism Quotient (AQ, averaging items 61–65, not included in the Total Score) [30].

*The Sensory Perception Quotient—Short Form 35 item (SPQ-SF35).* It is a 35-item self-report questionnaire investigating hyper- or hyposensitivity in the five modes of perception, able to discriminate between adults with ASD and NA. A Total Score and five subscales (Vision, Taste, Smell, Touch, Hearing) were calculated, with higher scores meaning higher sensory threshold, thus suggesting a pattern of hyposensitivity in the sensory domain assessed (e.g. “I would notice if someone added 5 grains of salt to my cup of water”) [40].

### Statistical analyses

Statistical analyses were conducted with SPSS (Statistical Package for Social Science), version 28.

First, descriptive statistics were calculated for all the sociodemographic and clinical variables, and the prevalence of patients scoring above the cut-off at the EAT-26 was analyzed. Secondly, a series of two-stage linear regression models were conducted, with the EAT-26 and the SWEAA (Total Scores and subscales) as dependent variables and entering the following as independent variables: (i) Model 1: SPQ-SF35 Subscales. (ii) Model 2: SPQ-SF35 Subscales, Age, and RAADS-R Total Score, in order to assess the impact of autistic traits’ severity on the association between sensory sensitivity and dysfunctional eating behaviours.

## Results

### Sociodemographic information

The mean age of our sample was 19.63 years (± 1.98). Most of our participants (156, 60.23%) identified themselves with the female gender, 86 (33.20%) with the male gender, 12 (4.63%) declared themselves non-binary, and 5 (1.93%) preferred not to disclose their gender. The average BMI was 22.62 kg/h^2^ (± 5.17): in particular, 48 (18.5%) participants were underweight (i.e., BMI below 18.5 kg/h^2^), 150 (57%) had a healthy weight (BMI included between 18.5 and 24.9 kg/h^2^), 40 (15.4%) participants were overweight (BMI included between 25 and 29.9 kg/h^2^) and 21 (8.1%) participants were obese (BMI above 30 kg/h^2^).

The clinician, at the end of the first consultation, categorized their symptomatology as follows: 104 participants presented an anxiety symptomatology; 56 participants a personality disorders symptomatology; 26 presented potential mood disorders; 8 showed disorders with onset during childhood or neurodevelopmental condition; 4 showed psychotic symptoms; 3 eating behavior related symptoms; 54 showed multiple symptoms (among which 2 subjects had a possible neurodevelopmental condition), to be further investigated.

Further information is reported in Table 1.

**Table 1:** Sociodemographic and psychometric features

	Value
Age, mean (SD)	19.63 (1.98)
BMI, mean (SD)	22.62 (2.17)
BMI, N (%)	
Underweight	48 (18.5)
Healthy weight	150 (57.9)
Overweight	40 (15.4)
Obese	21 (8.1)
Gender, N (%)	
Female	156 (60.23)
Male	86 (33.20)
Not binary	12 (4.63)
Undeclared	5 (1.93)
Education	
Middle School	91 (35.14)
3-year professional licence	19 (7.34)
Diploma	139 (53.67)
Bachelor degree	9 (3.47)
Master degree	1 (0.39)
Employment	
Student	195 (75.29)
Employed	34 (13.13)
Unemployed	30 (11.58)
Living condition	
Living alone	5 (1.93)
Living with parents	232 (89.58)
Living with partner	5 (1.93)
Living with flatmates	15 (5.79)
Living in a therapeutic community	2 (0.77)
AQ Total Score, mean (SD)	22.08 (7.94)
AQ Total Score, N (%)	
Below cut-off	217 (83.78)
Above cut-off	42 (16.22)
AQ Social skills, mean (SD)	4.18 (2.61)
AQ Attention switching, mean (SD)	5.98 (2.19)
AQ Attention to detail, mean (SD)	4.77 (2.38)
AQ Comunication, mean (SD)	3.73 (2.45)
AQ Imagination, mean (SD)	3.40 (1.93)
RAADS-R Total Score, mean (SD)	82.33 (45.21)
RAADS-R Total Score, N (%)	
Below cut-off	110 (42.47)
Above cut-off	149 (57.53)
RAADS-R Social Relatedness, mean (SD)	39.98 (21.40)
RAADS-R Circumscribed Interests, mean (SD)	16.82 (10.16)
RAADS-R Language, mean (SD)	6.10 (4.64)
RAADS-R Sensory-motor, mean (SD)	19.43 (14.52)
EAT-26 Total Score, mean (SD)	12.96 (13.29)
EAT-26 Total Score, N (%)	
Below cut-off	198 (76.45)
Above cut-off	61 (23.55)
EAT-26 Dieting, mean (SD)	7.23 (8.06)
EAT-26 Bulimia and Food Preoccupation, mean (SD)	2.56 (3.81)
EAT-26 Oral Control, mean (SD)	3.17 (3.71)
SWEAA Total Score, mean (SD)	0.98 (0.48)
SWEAA Perception, mean (SD)	1.27 (0.79)
SWEAA Motor Control, mean (SD)	0.66 (0.53)
SWEAA Purchase of Food, mean (SD)	1.37 (1.01)
SWEAA Eating Behaviour, mean (SD)	1.08 (0.71)
SWEAA Mealtime Surrounding, mean (SD)	0.98 (0.78)
SWEAA Social Situation at Mealtime, mean (SD)	1.34 (0.55)
SWEAA Other Behaviour Associated with Disturbed Eating, mean (SD)	0.40 (0.45)
SWEAA Hunger/Satiety, mean (SD)	0.86 (0.81)
SWEAA Simultaneous Capacity, mean (SD)	0.53 (1.01)
SWEAA Pica, mean (SD)	0.10 (0.47)
SPQ-SF35 Total Score, mean (SD)	56.24 (17.55)
SPQ-SF35 Vision, mean (SD)	10.64 (3.87)
SPQ-SF35 Smell, mean (SD)	16.45 (5.49)
SPQ-SF35 Taste, mean (SD)	5.33 (2.74)
SPQ-SF35 Touch, mean (SD)	14.77 (5.65)
SPQ-SF35 Hearing, mean (SD)	9.05 (3.22)

*AQ*  Autism Quotient, *BMI*  Body Mass Index, *EAT-26*  Eating Attitude Test—26 items, *N*  numerosity, *RAADS-R*  Ritvo Autism Asperger diagnostic scale—revised, *SD*  standard deviation, *SPQ-SF35*  Sensory Perception Quotient—Short Form 35 items, *SWEAA*  Swedish Eating Assessment for Autism Spectrum Disorders

### Psychometric information

At the EAT-26, 61 (23.55%) participants scored above the cut-off, suggesting that they presented a risk for eating disorders and should be assessed by a specialized clinician. The mean score at the EAT-26 *Total Score* was 12.96 (± 13.29); at the SWEAA *Total Score* it was 0.98 (± 0.48), and at the SPQ-SF35 *Total Score* it was 56.24 (± 17.55). With respect to the autistic traits, 42 participants (16.22%) scored above the cut-off at the AQ, and 149 (57.53%) participants scored above the cut-off at the RAADS-R. Further results are reported in Table 1.

### EAT‑26

In model 2, controlling for participants’ age and severity of autistic traits (RAADS-R), SPQ-SF35 *Touch* predicted the EAT-26 *Total Score* (b = − 0.519, p = 0.031); a trend towards significance emerged at the subscales *Dieting* (b = − 0.289, p = 0.052), and *Bulimia and Food Preoccupation* (b = − 0.124, p = 0.078). The RAADS-R was significantly associated with the EAT-26 *Total Score* (b = 0.061 p = 0.006) and its subscale *Oral Control* (b = 0.026, p < 0.001); a trend towards significance emerged at the subscale *Bulimia and Food Preoccupation* (b = 0.012, p = 0.053). Age was not associated with any of the EAT-26 scores. The R^2^ always increased from Model 1 to Model 2, with a difference comprised between 0.014 (EAT-26 *Dieting*) and 0.066 (EAT-26 *Oral Control*). Further details are reported in Supplementary material.

### SWEAA

In model 2, controlling for participants’ age and severity of autistic traits (RAADS-R), SPQ-SF35 *Vision* predicted the SWEAA *Total Score* (b = − 0.02, p = 0.026) and its subscales *Perception* (b = − 0.04, p = 0.011), *Motor Control* (b = -0.025, p = 0.046) and *Social situation at mealtime* (b = − 0.029, p = 0.023). At the SPQ-SF35 *Smell,* a trend towards significance emerged with the variable *Pica* (b = − 0.015, p = 0.052). SPQ-SF35 *Touch* predicted the SWEAA subscale *Social situation at mealtime* (b = 0.019, p = 0.038). The RAADS-R was significantly associated with the SWEAA *Total Score* (b = 0.006, p < 0.001) and its subscales *Perception* (b = 0.008, p < 0.001), *Motor Control* (b = 0.005, p < 0.001), *Purchase of food* (b = 0.005, p = 0.003), *Eating behaviour* (b = 0.006, p < 0.001), *Mealtime surrounding* (b = 0.008, p < 0.001), *Social situation at mealtime* (b = 0.004, p < 0.001), *Other behaviour associated with disturbed eating* (b = 0.002, p = 0.033), *Hunger/Satiety* (b = 0.004, p = 0.001), *Simultaneous Capacity* (b = 0.009, p < 0.001), *Pica* (b = 0.003, p = 0.001). The age was significantly associated with the SWEAA subscales *Perception* (b = − 0.039, p = 0.039), *Social situation at mealtime* (b = − 0.035, p = 0.025). The R^2^ always increased from Model 1 to Model 2, with a difference comprised between 0.035 (SWEAA *Purchase of Food*) and 0.2 (SWEAA *Total Score*). Further details are reported in Supplementary Materials.

## Discussion

The aim of this study was to investigate the prevalence of symptomatology suggestive of an eating disorder, and its relationship with sensory sensitivity and the severity of autistic traits, in a group of young adults who referred to a mental health outpatient clinic for different forms of psychological distress and had not received a formal diagnosis yet.

First, we found that, among our participants, 23.55% scored above the cut-off at the EAT-26, suggesting that they presented a potential risk for eating disorders. Secondly, we observed that altered sensory sensitivity in vision and touch domains was associated with a higher occurrence of autistic-like eating behaviors (SWEAA) and general eating disorder symptomatology (EAT-26). Finally, the presence of the RAADS-R in our statistical model significantly contributed to explaining the variance not only at SWEAA score, which was predicted since the SWEAA was specifically validated to investigate autistic-like eating behaviours, but also at the EAT-26 scores, a screening measure for eating disorders symptoms.

### The incidence of eating disorders risk

Among our participants, 23.55% scored above the cut-off at the EAT-26, suggesting that they presented a potential risk for eating disorders, hence requiring a further assessment by a specialized clinician. Such a wide spreading of abnormal feeding and eating behaviours within an unselected cohort of patients aligns with the established notion that these behaviours may emerge as secondary manifestations of underlying psychological and psychiatric disturbances, such as anxiety and mood disorders, which frequently contribute to maladaptive eating patterns [41]. The utilization of food as a coping mechanism, a tool for regulating emotions [42], and a mean to gain acceptance within peer groups [43], whether for health or aesthetic reasons [44], has been well-documented among adolescents and young adults. Our data further underscore the significance of the feeding and eating domain as a central psychopathological feature in the distress experienced by young adults with psychiatric symptoms and psychological suffering.

### The relationship between sensory sensitivity, autism-related eating behaviours, and ED symptomatology

A heightened sensory sensitivity in the vision domain and a hyposensitivity in the touch domain appeared associated with a higher presence of autistic-like eating behaviours, as per SWEAA; on the other hand, a hypersensitivity in the touch domain was associated with the Total Score of the EAT-26, accounting for general ED symptomatology, with a trend towards significance in its subscale Dieting. This discrepancy in the role of touch, and, specifically, its hypo- and hyper-sensitivity respectively, might be due to the specific phenomena that the two subscales investigate: at the SWEAA, the sensitivity to touch was related to the subscale “Social situation at mealtime”, which investigates items such as “I eat in my bedroom”, “I eat with the ones I live”, “I leave the table as soon as the food is eaten”; in the EAT-26, touch was involved in the Total Score, accounting for general ED symptomatology, and in the subscale Dieting, assessing the attention to calories ingested and burned doing physical exercise, the desire to be thin, etc. (e.g. “I feel extremely guilty after eating”). The RAADS-R was significantly associated with the SWEAA *Total Score* and most of its subscales; this was not surprising considering the SWEAA was specifically validated to investigate autistic-like eating behaviours. Moreover, it was expected that the R^2^, which is considered an index of “good fit” of the model to the real data, would have increased when inserting the RAADS-R in our regression analysis: as a matter of fact, the R^2^ always increased from Model 1 to Model 2, with a difference comprised between the 3.5% (SWEAA *Purchase of Food*) to the 20% (SWEAA *Total Score*). Most importantly, the RAADS-R was also significantly associated with the EAT-26 *Total Score* and its subscale *Oral Control*; again, when adding the RAADS-R to our model, the R^2^ always increased from Model 1 to Model 2, with a difference comprised between 1.4% (EAT-26 *Dieting*) and 6.6% (EAT-26 *Oral Control*). In other words, the presence of autistic traits in our sample significantly contributed to better explain part of the variance not just within the SWEAA, which is based on autistic-like behaviours per se, but also within the EAT-26, which assesses general eating symptomatology. We previously found that this was true in a population with diagnosed ASD [34] and in a group of patients diagnosed with ED [35]; with this study, we make a step further by finding this significant relationship also in the current population, consisting of young adults manifesting general psychopathological distress, without a formal diagnosis yet. With respect to the autistic traits per se, it should be considered that 42 participants (16.22%) scored above the cut-off at the AQ, and 149 (57.53%) participants scored above the cut-off at the RAADS-R; further results and considerations about autistic traits in the same population are reported elsewhere [45]. Overall, our findings align with recent literature indicating a robust association between autistic traits, especially when considered along a spectrum or in a continuous form, including subthreshold traits, sensory sensitivity, and eating disturbances. For instance, Young et al. [46] reported a significant association between sensory sensitivity and the Eating Disorder Examination Questionnaire, another screening measure for eating disorders, in individuals with ASD. Furthermore, Brede et al. [6] conducted an extensive qualitative study involving women with ASD and restrictive AN, discovering that sensory sensitivities contributed to the development of eating disorders in women with ASD. This contribution occurred through general sensory overload, food-specific sensory sensitivities, and discomfort and confusion related to internal and bodily sensations, ultimately leading one participant to state: "For me, anorexia is just a symptom, and the cause is autism" ([6], p. 4288).

### Strength and limits

The strengths of our study mainly consist in the large sample size, composed by adolescents and young adults who referred for the first time to a generalized mental health outpatient clinic. Moreover, our study was built on previous studies conducted by our group and our results are in line with other clinical studies reported in the recent literature on the topic.

Our study presents the following limitations: first, all data were collected through self-reported questionnaires; although self-report measures are thought to provide a more accurate description of the patient’s experience, we cannot rule out the possibility that our patients might have emphasized symptoms and signs of their discomfort, given the condition of struggle and distress that they were living with at the moment of testing; future perspectives include replicating these findings in study protocols that incorporate clinician-rated clinical scales, in order to account for potential distortions. Second, the restricted age range of our population may affect the generalizability of the result; future studies should investigate whether the results obtained so far can be validated in a population experiencing psychiatric and psychological distress at a later stage in life. Third, at the moment of writing, we did not have a formal diagnosis of each patient yet, hence other psychiatric conditions that may mediate or explain the presence of autistic traits in the study sample have not been considered. Analogous analyses should be conducted with a focus on exploring the relationship between ASD, autistic traits, and other psychopathological domains and categories, to provide insights into the potential associations between subthreshold autistic traits and other major psychiatric conditions. As a matter of fact, our findings open to a series of new research question: is the presence of autistic traits, even subthreshold, a proper mediating factor between a physiological alteration in sensory sensitivity and the development of dysfunctional eating behaviours? Is this relationship specific to ASD and ED, or could it be considered as a subthreshold trait, that might be generalized and becomes evident in other psychiatric conditions (differently categorized) during acute suffering episodes? If this is the case, which factors can be considered as potentially precipitating?

## Conclusions

In conclusion, first, we found that among a population of young adults referring for the first time to a mental health outpatient clinic for generalized psychiatric and psychological distress, almost a quarter of patients (23.55%) scored above the cut-off at the EAT-26, hence requiring a further assessment for eating disorders by a specialized clinician. This underscores the significance of the eating domain as a central psychopathological feature in the distress experienced by young adults.

Second, our results add evidence to the possible association between sensory sensitivity, autistic traits and eating disorders, outlining that a putative substrate for the development of abnormal eating behaviours could be represented by the relationship between some degree of distortion of the intensity of sensorial information and the presence of potentially subthreshold autistic traits.

### What is already known on this subject?

In addition to other psychopathological features such as cognitive rigidity, atypical social cognition and difficulties in emotion processing, both adults with Autism Spectrum Disorders (ASD) and adults with eating disorder (ED) showed sensory sensitivity anomalies, which resulted associated with eating disturbances. These findings prompt further exploration into the connection between autistic traits (even subthreshold ones), sensory sensitivity, and dysfunctional eating behaviors across psychiatric conditions, aligning with a dimensional approach to psychopathology.

### What does this study add?

Results from our study indicate that a quarter of our participants, composed by 259 patients who were referred for the first time to a mental health outpatient clinic, exhibited a potential risk for eating disorders, warranting assessment by specialized clinicians; these findings underscore the pivotal role of the eating domain as a central psychopathological feature in the distress faced by young adults. Moreover, the presence of autistic traits in our sample significantly contributed to explain the results at two screening questionnaires, investigating autistic-like behaviours and general eating symptomatology. Hence, we provided further evidence to the association between sensory sensitivity, autistic traits and eating disorders, prompting new research inquiries into the role of subthreshold autistic traits in general psychopathology.

### Supplementary Information

Below is the link to the electronic supplementary material.

Supplementary file1 (DOCX 100 KB)

## Data Availability

Anonymized data will be shared by request from any qualified investigator. No datasets were generated or analysed during the current study.
