# Supplementary material for: Autistic traits, sensory sensitivity and eating disturbances in a sample of young adults referring to a generalized mental health clinic
Source: Eat Weight Disord. 2024 Jan 23;29(1):10. doi: 10.1007/s40519-024-01639-7 (PMC10806192; doi:10.1007/s40519-024-01639-7)
Supplement: Supplementary file 1 — Supplementary file1 (DOCX 100 KB) [file 40519_2024_1639_MOESM1_ESM.docx]

**Supplementary Materials**

Complete results of the two-stage linear regression models, with the EAT-26 and the SWEAA (Total Scores and subscales, separately) as dependent variables, and the following as independent variables: (i) Model 1: SPQ-SF35 Subscales; (ii) Model 2: SPQ-SF35 Subscales, Age, RAADS-R Total Score. The inclusion of the RAADS-R measures the impact of the severity of the autistic traits.

Abbreviations: EAT-26 = Eating Attitude Test – 26 items; RAADS-R = Ritvo Autism Asperger Diagnostic Scale-Revised; SPQ-SF35 = Sensory Perception Quotient – Short Form (35 items); SWEAA = Swedish Eating Assessment for Autism Spectrum Disorders.

Bold values: p < 0.05

***EAT-26***

In model 1, SPQ-SF35 *Touch* predicted the EAT-26 *Total Score* (b = -0.575, p = 0.018; a trend towards significance emerged at the subscale *Dieting* (b = -0.289, p = 0.052), *Bulimia and Food Preoccupation* (b = -0.124, p = 0.078). Model 2 is described in the main text.

| EAT-26 Total Score | | | | | | | | | | |
| --- | --- | --- | --- | --- | --- | --- | --- | --- | --- | --- |
| Model |  | B | Standard error | Standardized coefficient - Beta | t | p | 95% C.I. Inf. Limit | 95% C.I. Sup. Limit | R^2^ | Durbin-Watson |
| 1 | Intercept | 20.366 | 2.812 |  |  | < 0.001 | 14.828 | 25.904 | 0.058 |  |
|  | SPQ-SF Vision | -0.361 | 0.328 | -0.105 | -0.105 | 0.273 | -1.008 | 0.286 |  |  |
|  | SPQ-SF Smell | 0.039 | 0.212 | 0.016 | 0.016 | 0.856 | -0.379 | 0.456 |  |  |
|  | SPQ-SF Taste | 0.542 | 0.400 | 0.112 | 0.112 | 0.176 | -0.245 | 1.330 |  |  |
|  | SPQ-SF Touch | -0.575 | 0.241 | -0.245 | -0.245 | 0.018 | -1.049 | -0.101 |  |  |
|  | SPQ-SF Hearing | 0.155 | 0.413 | 0.038 | 0.038 | 0.708 | -0.659 | 0.968 |  |  |
| 2 | Intercept | 18.726 | 9.083 |  |  | 0.040 | 0.837 | 36.616 | 0.090 | 2.032 |
|  | SPQ-SF Vision | -0.088 | 0.338 | -0.026 | -0.026 | 0.795 | -0.753 | 0.577 |  |  |
|  | SPQ-SF Smell | -0.082 | 0.213 | -0.034 | -0.034 | 0.701 | -0.502 | 0.338 |  |  |
|  | SPQ-SF Taste | 0.650 | 0.396 | 0.134 | 0.134 | 0.102 | -0.130 | 1.430 |  |  |
|  | SPQ-SF Touch | -0.519 | 0.240 | -0.221 | -0.221 | 0.031 | -0.991 | -0.046 |  |  |
|  | SPQ-SF Hearing | 0.377 | 0.415 | 0.091 | 0.091 | 0.365 | -0.441 | 1.194 |  |  |
|  | RAADS-R | 0.061 | 0.022 | 0.208 | 0.208 | 0.006 | 0.018 | 0.104 |  |  |
|  | Age | -0.394 | 0.409 | -0.059 | -0.059 | 0.337 | -1.200 | 0.412 |  |  |

| EAT-26 Dieting | | | | | | | | | | |
| --- | --- | --- | --- | --- | --- | --- | --- | --- | --- | --- |
| Model |  | B | Standard error | Standardized coefficient - Beta | t | p | 95% C.I. Inf. Limit | 95% C.I. Sup. Limit | R^2^ | Durbin-Watson |
| 1 | Intercept | 10.316 | 1.723 |  | 5.987 | < 0.001 | 6.923 | 13.709 | 0.037 |  |
|  | SPQ-SF Vision | -0.214 | 0.201 | -0.103 | -1.064 | 0.288 | -0.610 | 0.182 |  |  |
|  | SPQ-SF Smell | -0.010 | 0.130 | -0.007 | -0.077 | 0.939 | -0.266 | 0.246 |  |  |
|  | SPQ-SF Taste | 0.360 | 0.245 | 0.122 | 1.470 | 0.143 | -0.122 | 0.843 |  |  |
|  | SPQ-SF Touch | -0.306 | 0.147 | -0.215 | -2.078 | 0.039 | -0.597 | -0.016 |  |  |
|  | SPQ-SF Hearing | 0.217 | 0.253 | 0.087 | 0.857 | 0.393 | -0.282 | 0.715 |  |  |
| 2 | Intercept | 11.373 | 5.622 |  | 2.023 | 0.044 | 0.301 | 22.445 | 0.051 | 1.985 |
|  | SPQ-SF Vision | -0.111 | 0.209 | -0.053 | -0.531 | 0.596 | -0.522 | 0.301 |  |  |
|  | SPQ-SF Smell | -0.057 | 0.132 | -0.039 | -0.434 | 0.664 | -0.317 | 0.202 |  |  |
|  | SPQ-SF Taste | 0.402 | 0.245 | 0.137 | 1.641 | 0.102 | -0.081 | 0.885 |  |  |
|  | SPQ-SF Touch | -0.289 | 0.148 | -0.203 | -1.951 | 0.052 | -0.582 | 0.003 |  |  |
|  | SPQ-SF Hearing | 0.310 | 0.257 | 0.124 | 1.206 | 0.229 | -0.196 | 0.816 |  |  |
|  | RAADS-R | 0.023 | 0.014 | 0.127 | 1.669 | 0.096 | -0.004 | 0.049 |  |  |
|  | Age | -0.232 | 0.253 | -0.057 | -0.916 | 0.360 | -0.731 | 0.267 |  |  |

| EAT-26 Bulimia and Food Preoccupation | | | | | | | | | | |
| --- | --- | --- | --- | --- | --- | --- | --- | --- | --- | --- |
| Model |  | B | Standard error | Standardized coefficient - Beta | t | p | 95% C.I. Inf. Limit | 95% C.I. Sup. Limit | R^2^ | Durbin-Watson |
| 1 | Intercept | 4.437 | 0.812 |  | 5.463 | < 0.001 | 2.838 | 6.037 | 0.042 |  |
|  | SPQ-SF Vision | -0.103 | 0.095 | -0.105 | -1.085 | 0.279 | -0.290 | 0.084 |  |  |
|  | SPQ-SF Smell | 0.013 | 0.061 | 0.018 | 0.209 | 0.835 | -0.108 | 0.133 |  |  |
|  | SPQ-SF Taste | 0.107 | 0.116 | 0.077 | 0.929 | 0.354 | -0.120 | 0.335 |  |  |
|  | SPQ-SF Touch | -0.132 | 0.070 | -0.197 | -1.905 | 0.058 | -0.269 | 0.004 |  |  |
|  | SPQ-SF Hearing | 0.044 | 0.119 | 0.037 | 0.365 | 0.715 | -0.191 | 0.279 |  |  |
| 2 | Intercept | 5.198 | 2.642 |  | 1.967 | 0.050 | -0.006 | 10.402 | 0.062 | 2.131 |
|  | SPQ-SF Vision | -0.046 | 0.098 | -0.047 | -0.469 | 0.639 | -0.239 | 0.147 |  |  |
|  | SPQ-SF Smell | -0.013 | 0.062 | -0.019 | -0.216 | 0.829 | -0.135 | 0.109 |  |  |
|  | SPQ-SF Taste | 0.131 | 0.115 | 0.094 | 1.133 | 0.258 | -0.096 | 0.358 |  |  |
|  | SPQ-SF Touch | -0.124 | 0.070 | -0.183 | -1.772 | 0.078 | -0.261 | 0.014 |  |  |
|  | SPQ-SF Hearing | 0.096 | 0.121 | 0.081 | 0.792 | 0.429 | -0.142 | 0.334 |  |  |
|  | RAADS-R | 0.012 | 0.006 | 0.147 | 1.945 | 0.053 | < 0.001 | 0.025 |  |  |
|  | Age | -0.137 | 0.119 | -0.071 | -1.147 | 0.252 | -0.371 | 0.098 |  |  |

| EAT-26 Oral Control | | | | | | | | | | |
| --- | --- | --- | --- | --- | --- | --- | --- | --- | --- | --- |
| Model |  | B | Standard error | Standardized coefficient - Beta | t | p | 95% C.I. Inf. Limit | 95% C.I. Sup. Limit | R^2^ | Durbin-Watson |
| 1 | Intercept | 5.613 | 0.782 |  | 7.181 | < 0.001 | 4.073 | 7.152 | 0.067 |  |
|  | SPQ-SF Vision | -0.044 | 0.091 | -0.046 | -0.479 | 0.632 | -0.223 | 0.136 |  |  |
|  | SPQ-SF Smell | 0.036 | 0.059 | 0.053 | 0.607 | 0.545 | -0.080 | 0.152 |  |  |
|  | SPQ-SF Taste | 0.075 | 0.111 | 0.055 | 0.673 | 0.502 | -0.144 | 0.294 |  |  |
|  | SPQ-SF Touch | -0.136 | 0.067 | -0.207 | -2.037 | 0.043 | -0.268 | -0.005 |  |  |
|  | SPQ-SF Hearing | -0.106 | 0.115 | -0.092 | -0.921 | 0.358 | -0.332 | 0.120 |  |  |
| 2 | Intercept | 2.155 | 2.476 |  | 0.870 | 0.385 | -2.721 | 7.031 | 0.133 | 1.957 |
|  | SPQ-SF Vision | 0.069 | 0.092 | 0.072 | 0.752 | 0.452 | -0.112 | 0.250 |  |  |
|  | SPQ-SF Smell | -0.011 | 0.058 | -0.017 | -0.195 | 0.846 | -0.126 | 0.103 |  |  |
|  | SPQ-SF Taste | 0.117 | 0.108 | 0.086 | 1.085 | 0.279 | -0.096 | 0.330 |  |  |
|  | SPQ-SF Touch | -0.106 | 0.065 | -0.161 | -1.616 | 0.107 | -0.234 | 0.023 |  |  |
|  | SPQ-SF Hearing | -0.029 | 0.113 | -0.025 | -0.255 | 0.799 | -0.252 | 0.194 |  |  |
|  | RAADS-R | 0.026 | 0.006 | 0.317 | 4.369 | < 0.001 | 0.014 | 0.038 |  |  |
|  | Age | -0.025 | 0.112 | -0.013 | -0.223 | 0.824 | -0.245 | 0.195 |  |  |

***SWEAA***

In model 1, SPQ-SF35 *Vision* predicted the SWEAA *Total Score* (b = - 0.045, p < 0.001) and its subscales *Perception* (b = -0.076, p < 0.001), *Motor Control* (b = -0.045, p < 0.001), *Purchase of food* (b = -0.054, p = 0.023), *Eating Behaviour* (b = -0.041, p = 0.015), *Mealtime surrounding* (b = -0.038, p = 0.032), *Social situation at mealtime* (b = -0.048, p < 0.001), *Simultaneous capacity* (b = -0.065, p = 0.009), and a trend towards significance emerged with the variable *Pica* (b = -0.023, p = 0.051). SPQ-SF35 *Smell* predicted the SWEAA *Total Score* (b = 0.013, p = 0.046) and its subscales *Motor control* (b = 0.017, p = 0.040), *Social situation at mealtime* (b = 0.022, p = 0.009), *Simultaneous capacity* (b = 0.032, p = 0.040), and a trend towards significance emerged with the variable *Hunger/Satiety* (b = 0.023, p = 0.068). SPQ-SF35 *Touch* predicted the SWEAA subscale Hunger/Satiety (b = -0-03, p = 0,037) and a trend towards significance emerged with the variable *Perception* (b = -0.023, p = 0.057). SPQ-SF35 *Hearing* predicted the SWEAA *Total Score* (b = - 0.03, p = 0.017) and its subscales *Mealtime surrounding* (b = -0.051, p = 0.022), *Social situation at mealtime* (b = -0.044, p = 0.007) and *Simultaneous capacity* (b = -0.072, p = 0.019). Model 2 is described in the main text.

| SWEAA Total Score | | | | | | | | | | |
| --- | --- | --- | --- | --- | --- | --- | --- | --- | --- | --- |
| Model |  | B | Standard error | Standardized coefficient - Beta | t | p | 95% C.I. Inf. Limit | 95% C.I. Sup. Limit | R^2^ | Durbin-Watson |
| 1 | Intercept | 1.708 | 0.086 |  | 19.813 | < 0.001 | 1.539 | 1.878 | 0.313 |  |
|  | SPQ-SF Vision | -0.045 | 0.010 | -0.364 | -4.456 | < 0.001 | -0.065 | -0.025 |  |  |
|  | SPQ-SF Smell | 0.013 | 0.007 | 0.150 | 2.002 | 0.046 | < 0.001 | 0.026 |  |  |
|  | SPQ-SF Taste | -0.011 | 0.012 | -0.062 | -0.876 | 0.382 | -0.035 | 0.013 |  |  |
|  | SPQ-SF Touch | -0.009 | 0.007 | -0.106 | -1.211 | 0.227 | -0.023 | 0.006 |  |  |
|  | SPQ-SF Hearing | -0.030 | 0.013 | -0.205 | -2.401 | 0.017 | -0.055 | -0.005 |  |  |
| 2 | Intercept | 1.222 | 0.239 |  | 5.107 | < 0.001 | 0.751 | 1.693 | 0.511 | 2.142 |
|  | SPQ-SF Vision | -0.020 | 0.009 | -0.161 | -2.242 | 0.026 | -0.037 | -0.002 |  |  |
|  | SPQ-SF Smell | 0.002 | 0.006 | 0.027 | 0.418 | 0.677 | -0.009 | 0.013 |  |  |
|  | SPQ-SF Taste | -0.001 | 0.010 | -0.007 | -0.111 | 0.911 | -0.022 | 0.019 |  |  |
|  | SPQ-SF Touch | -0.003 | 0.006 | -0.034 | -0.458 | 0.647 | -0.015 | 0.010 |  |  |
|  | SPQ-SF Hearing | -0.012 | 0.011 | -0.081 | -1.094 | 0.275 | -0.033 | 0.010 |  |  |
|  | RAADS-R | 0.006 | 0.001 | 0.537 | 9.848 | < 0.001 | 0.005 | 0.007 |  |  |
|  | Age | -0.019 | 0.011 | -0.080 | -1.784 | 0.076 | -0.040 | 0.002 |  |  |

| SWEAA Perception | | | | | | | | | | |
| --- | --- | --- | --- | --- | --- | --- | --- | --- | --- | --- |
| Model |  | B | Standard error | Standardized coefficient - Beta | t | p | 95% C.I. Inf. Limit | 95% C.I. Sup. Limit | R^2^ | Durbin-Watson |
| 1 | Intercept | 2.496 | 0.143 |  | 17.427 | < 0.001 | 2.214 | 2.779 | 0.306 |  |
|  | SPQ-SF Vision | -0.076 | 0.017 | -0.373 | -4.545 | < 0.001 | -0.109 | -0.043 |  |  |
|  | SPQ-SF Smell | 0.013 | 0.011 | 0.088 | 1.167 | 0.244 | -0.009 | 0.034 |  |  |
|  | SPQ-SF Taste | -0.009 | 0.020 | -0.031 | -0.435 | 0.664 | -0.049 | 0.031 |  |  |
|  | SPQ-SF Touch | -0.023 | 0.012 | -0.168 | -1.909 | 0.057 | -0.048 | 0.001 |  |  |
|  | SPQ-SF Hearing | -0.026 | 0.021 | -0.106 | -1.233 | 0.219 | -0.067 | 0.016 |  |  |
| 2 | Intercept | 1.996 | 0.414 |  | 4.824 | < 0.001 | 1.181 | 2.811 | 0.464 | 1.896 |
|  | SPQ-SF Vision | -0.040 | 0.015 | -0.194 | -2.569 | 0.011 | -0.070 | -0.009 |  |  |
|  | SPQ-SF Smell | -0.003 | 0.010 | -0.022 | -0.333 | 0.740 | -0.022 | 0.016 |  |  |
|  | SPQ-SF Taste | 0.005 | 0.018 | 0.019 | 0.296 | 0.767 | -0.030 | 0.041 |  |  |
|  | SPQ-SF Touch | -0.015 | 0.011 | -0.108 | -1.385 | 0.167 | -0.037 | 0.006 |  |  |
|  | SPQ-SF Hearing | 0.002 | 0.019 | 0.009 | 0.118 | 0.906 | -0.035 | 0.039 |  |  |
|  | RAADS-R | 0.008 | 0.001 | 0.473 | 8.272 | < 0.001 | 0.006 | 0.010 |  |  |
|  | Age | -0.039 | 0.019 | -0.097 | -2.076 | 0.039 | -0.075 | -0.002 |  |  |

| SWEAA Motor Control | | | | | | | | | | |
| --- | --- | --- | --- | --- | --- | --- | --- | --- | --- | --- |
| Model |  | B | Standard error | Standardized coefficient - Beta | t | p | 95% C.I. Inf. Limit | 95% C.I. Sup. Limit | R^2^ | Durbin-Watson |
| 1 | Intercept | 1.152 | 0.107 |  | 10.801 | < 0.001 | 0.942 | 1.362 | 0.156 |  |
|  | SPQ-SF Vision | -0.045 | 0.012 | -0.324 | -3.584 | < 0.001 | -0.069 | -0.020 |  |  |
|  | SPQ-SF Smell | 0.017 | 0.008 | 0.171 | 2.065 | 0.040 | 0.001 | 0.032 |  |  |
|  | SPQ-SF Taste | 0.003 | 0.015 | 0.017 | 0.220 | 0.826 | -0.027 | 0.033 |  |  |
|  | SPQ-SF Touch | -0.006 | 0.009 | -0.067 | -0.687 | 0.493 | -0.024 | 0.012 |  |  |
|  | SPQ-SF Hearing | -0.024 | 0.016 | -0.144 | -1.520 | 0.130 | -0.055 | 0.007 |  |  |
| 2 | Intercept | 0.533 | 0.329 |  | 1.622 | 0.106 | -0.114 | 1.180 | 0.258 | 2.05 |
|  | SPQ-SF Vision | -0.025 | 0.012 | -0.178 | -2.007 | 0.046 | -0.049 | < 0.001 |  |  |
|  | SPQ-SF Smell | 0.008 | 0.008 | 0.085 | 1.066 | 0.287 | -0.007 | 0.023 |  |  |
|  | SPQ-SF Taste | 0.011 | 0.014 | 0.056 | 0.760 | 0.448 | -0.017 | 0.039 |  |  |
|  | SPQ-SF Touch | -0.001 | 0.009 | -0.009 | -0.093 | 0.926 | -0.018 | 0.016 |  |  |
|  | SPQ-SF Hearing | -0.010 | 0.015 | -0.061 | -0.675 | 0.500 | -0.040 | 0.019 |  |  |
|  | RAADS-R | 0.005 | 0.001 | 0.394 | 5.868 | < 0.001 | 0.003 | 0.006 |  |  |
|  | Age | -0.004 | 0.015 | -0.016 | -0.291 | 0.771 | -0.033 | 0.025 |  |  |

| SWEAA Purchase of Food | | | | | | | | | | |
| --- | --- | --- | --- | --- | --- | --- | --- | --- | --- | --- |
| Model |  | B | Standard error | Standardized coefficient - Beta | t | p | 95% C.I. Inf. Limit | 95% C.I. Sup. Limit | R^2^ | Durbin-Watson |
| 1 | Intercept | 2.572 | 0.203 |  | 12.689 | < 0.001 | 2.172 | 2.971 | 0.149 |  |
|  | SPQ-SF Vision | -0.054 | 0.024 | -0.209 | -2.296 | 0.023 | -0.101 | -0.008 |  |  |
|  | SPQ-SF Smell | -0.008 | 0.015 | -0.045 | -0.545 | 0.586 | -0.038 | 0.022 |  |  |
|  | SPQ-SF Taste | -0.033 | 0.029 | -0.089 | -1.137 | 0.257 | -0.090 | 0.024 |  |  |
|  | SPQ-SF Touch | < 0.001 | 0.017 | 0.002 | 0.022 | 0.983 | -0.034 | 0.035 |  |  |
|  | SPQ-SF Hearing | -0.035 | 0.030 | -0.113 | -1.190 | 0.235 | -0.094 | 0.023 |  |  |
| 2 | Intercept | 1.029 | 0.652 |  | 1.579 | 0.116 | -0.255 | 2.313 | 0.184 | 1.946 |
|  | SPQ-SF Vision | -0.035 | 0.024 | -0.134 | -1.441 | 0.151 | -0.083 | 0.013 |  |  |
|  | SPQ-SF Smell | -0.015 | 0.015 | -0.084 | -1.012 | 0.312 | -0.046 | 0.015 |  |  |
|  | SPQ-SF Taste | -0.026 | 0.028 | -0.071 | -0.923 | 0.357 | -0.082 | 0.030 |  |  |
|  | SPQ-SF Touch | 0.008 | 0.017 | 0.046 | 0.473 | 0.637 | -0.026 | 0.042 |  |  |
|  | SPQ-SF Hearing | -0.027 | 0.030 | -0.087 | -0.917 | 0.360 | -0.086 | 0.031 |  |  |
|  | RAADS-R | 0.005 | 0.002 | 0.213 | 3.029 | 0.003 | 0.002 | 0.008 |  |  |
|  | Age | 0.043 | 0.029 | 0.084 | 1.455 | 0.147 | -0.015 | 0.101 |  |  |

| SWEAA Eating Behaviour | | | | | | | | | | |
| --- | --- | --- | --- | --- | --- | --- | --- | --- | --- | --- |
| Model |  | B | Standard error | Standardized coefficient - Beta | t | p | 95% C.I. Inf. Limit | 95% C.I. Sup. Limit | R^2^ | Durbin-Watson |
| 1 | Intercept | 1.688 | 0.145 |  | 11.648 | < 0.001 | 1.402 | 1.973 | 0.12 |  |
|  | SPQ-SF Vision | -0.041 | 0.017 | -0.226 | -2.451 | 0.015 | -0.075 | -0.008 |  |  |
|  | SPQ-SF Smell | 0.019 | 0.011 | 0.144 | 1.697 | 0.091 | -0.003 | 0.040 |  |  |
|  | SPQ-SF Taste | -0.012 | 0.021 | -0.046 | -0.584 | 0.560 | -0.053 | 0.029 |  |  |
|  | SPQ-SF Touch | -0.016 | 0.012 | -0.128 | -1.296 | 0.196 | -0.040 | 0.008 |  |  |
|  | SPQ-SF Hearing | -0.019 | 0.021 | -0.087 | -0.897 | 0.371 | -0.061 | 0.023 |  |  |
| 2 | Intercept | 1.055 | 0.451 |  | 2.342 | 0.020 | 0.168 | 1.943 | 0.212 | 2.055 |
|  | SPQ-SF Vision | -0.016 | 0.017 | -0.088 | -0.961 | 0.338 | -0.049 | 0.017 |  |  |
|  | SPQ-SF Smell | 0.008 | 0.011 | 0.060 | 0.739 | 0.461 | -0.013 | 0.029 |  |  |
|  | SPQ-SF Taste | -0.002 | 0.020 | -0.009 | -0.122 | 0.903 | -0.041 | 0.036 |  |  |
|  | SPQ-SF Touch | -0.010 | 0.012 | -0.076 | -0.804 | 0.422 | -0.033 | 0.014 |  |  |
|  | SPQ-SF Hearing | -0.001 | 0.021 | -0.005 | -0.051 | 0.960 | -0.042 | 0.040 |  |  |
|  | RAADS-R | 0.006 | 0.001 | 0.371 | 5.356 | < 0.001 | 0.004 | 0.008 |  |  |
|  | Age | -0.013 | 0.020 | -0.036 | -0.629 | 0.530 | -0.053 | 0.027 |  |  |

| SWEAA Mealtime Surrounding | | | | | | | | | | |
| --- | --- | --- | --- | --- | --- | --- | --- | --- | --- | --- |
| Model |  | B | Standard error | Standardized coefficient - Beta | t | p | 95% C.I. Inf. Limit | 95% C.I. Sup. Limit | R^2^ | Durbin-Watson |
| 1 | Intercept | 1.978 | 0.152 |  | 13.055 | < 0.001 | 1.680 | 2.276 | 0.201 |  |
|  | SPQ-SF Vision | -0.038 | 0.018 | -0.190 | -2.155 | 0.032 | -0.073 | -0.003 |  |  |
|  | SPQ-SF Smell | 0.012 | 0.011 | 0.086 | 1.068 | 0.287 | -0.010 | 0.035 |  |  |
|  | SPQ-SF Taste | -0.023 | 0.022 | -0.080 | -1.062 | 0.289 | -0.065 | 0.020 |  |  |
|  | SPQ-SF Touch | -0.014 | 0.013 | -0.102 | -1.079 | 0.282 | -0.040 | 0.012 |  |  |
|  | SPQ-SF Hearing | -0.051 | 0.022 | -0.212 | -2.297 | 0.022 | -0.095 | -0.007 |  |  |
| 2 | Intercept | 1.104 | 0.444 |  | 2.488 | 0.013 | 0.230 | 1.979 | 0.365 | 1.914 |
|  | SPQ-SF Vision | -0.001 | 0.016 | -0.005 | -0.058 | 0.953 | -0.033 | 0.032 |  |  |
|  | SPQ-SF Smell | -0.004 | 0.010 | -0.025 | -0.341 | 0.733 | -0.024 | 0.017 |  |  |
|  | SPQ-SF Taste | -0.009 | 0.019 | -0.031 | -0.450 | 0.653 | -0.047 | 0.029 |  |  |
|  | SPQ-SF Touch | -0.005 | 0.012 | -0.033 | -0.393 | 0.695 | -0.028 | 0.018 |  |  |
|  | SPQ-SF Hearing | -0.024 | 0.020 | -0.101 | -1.204 | 0.230 | -0.064 | 0.016 |  |  |
|  | RAADS-R | 0.008 | 0.001 | 0.494 | 7.947 | < 0.001 | 0.006 | 0.011 |  |  |
|  | Age | -0.021 | 0.020 | -0.054 | -1.065 | 0.288 | -0.061 | 0.018 |  |  |

| SWEAA Social Situation at Meatime | | | | | | | | | | |
| --- | --- | --- | --- | --- | --- | --- | --- | --- | --- | --- |
| Model |  | B | Standard error | Standardized coefficient - Beta | t | p | 95% C.I. Inf. Limit | 95% C.I. Sup. Limit | R^2^ | Durbin-Watson |
| 1 | Intercept | 1.757 | 0.111 |  | 15.864 | < 0.001 | 1.539 | 1.975 | 0.150 |  |
|  | SPQ-SF Vision | -0.048 | 0.013 | -0.336 | -3.699 | < 0.001 | -0.073 | -0.022 |  |  |
|  | SPQ-SF Smell | 0.022 | 0.008 | 0.219 | 2.639 | 0.009 | 0.006 | 0.038 |  |  |
|  | SPQ-SF Taste | -0.019 | 0.016 | -0.095 | -1.216 | 0.225 | -0.050 | 0.012 |  |  |
|  | SPQ-SF Touch | 0.015 | 0.009 | 0.158 | 1.628 | 0.105 | -0.003 | 0.034 |  |  |
|  | SPQ-SF Hearing | -0.044 | 0.016 | -0.258 | -2.707 | 0.007 | -0.076 | -0.012 |  |  |
| 2 | Intercept | 1.804 | 0.344 |  | 5.250 | < 0.001 | 1.127 | 2.480 | 0.243 | 2.137 |
|  | SPQ-SF Vision | -0.029 | 0.013 | -0.204 | -2.280 | 0.023 | -0.054 | -0.004 |  |  |
|  | SPQ-SF Smell | 0.014 | 0.008 | 0.135 | 1.689 | 0.092 | -0.002 | 0.029 |  |  |
|  | SPQ-SF Taste | -0.012 | 0.015 | -0.058 | -0.775 | 0.439 | -0.041 | 0.018 |  |  |
|  | SPQ-SF Touch | 0.019 | 0.009 | 0.194 | 2.084 | 0.038 | 0.001 | 0.037 |  |  |
|  | SPQ-SF Hearing | -0.028 | 0.016 | -0.163 | -1.780 | 0.076 | -0.059 | 0.003 |  |  |
|  | RAADS-R | 0.004 | 0.001 | 0.340 | 5.010 | < 0.001 | 0.003 | 0.006 |  |  |
|  | Age | -0.035 | 0.015 | -0.125 | -2.256 | 0.025 | -0.065 | -0.004 |  |  |

| SWEAA Other Behaviour Associated with Disturbed Eating | | | | | | | | | | |
| --- | --- | --- | --- | --- | --- | --- | --- | --- | --- | --- |
| Model |  | B | Standard error | Standardized coefficient - Beta | t | p | 95% C.I. Inf. Limit | 95% C.I. Sup. Limit | R^2^ | Durbin-Watson |
| 1 | Intercept | 0.700 | 0.096 |  | 7.326 | < 0.001 | 0.512 | 0.888 | 0.06 |  |
|  | SPQ-SF Vision | -0.012 | 0.011 | -0.104 | -1.088 | 0.278 | -0.034 | 0.010 |  |  |
|  | SPQ-SF Smell | 0.002 | 0.007 | 0.023 | 0.261 | 0.794 | -0.012 | 0.016 |  |  |
|  | SPQ-SF Taste | 0.014 | 0.014 | 0.082 | 0.995 | 0.321 | -0.013 | 0.040 |  |  |
|  | SPQ-SF Touch | -0.012 | 0.008 | -0.145 | -1.423 | 0.156 | -0.028 | 0.004 |  |  |
|  | SPQ-SF Hearing | -0.011 | 0.014 | -0.081 | -0.810 | 0.419 | -0.039 | 0.016 |  |  |
| 2 | Intercept | 0.756 | 0.310 |  | 2.436 | 0.016 | 0.145 | 1.367 | 0.082 | 1.954 |
|  | SPQ-SF Vision | -0.005 | 0.012 | -0.041 | -0.418 | 0.677 | -0.028 | 0.018 |  |  |
|  | SPQ-SF Smell | -0.001 | 0.007 | -0.018 | -0.199 | 0.842 | -0.016 | 0.013 |  |  |
|  | SPQ-SF Taste | 0.016 | 0.014 | 0.100 | 1.219 | 0.224 | -0.010 | 0.043 |  |  |
|  | SPQ-SF Touch | -0.010 | 0.008 | -0.130 | -1.268 | 0.206 | -0.027 | 0.006 |  |  |
|  | SPQ-SF Hearing | -0.005 | 0.014 | -0.035 | -0.344 | 0.731 | -0.033 | 0.023 |  |  |
|  | RAADS-R | 0.002 | 0.001 | 0.161 | 2.149 | 0.033 | < 0.001 | 0.003 |  |  |
|  | Age | -0.016 | 0.014 | -0.068 | -1.110 | 0.268 | -0.043 | 0.012 |  |  |

| SWEAA Hunger/Satiety | | | | | | | | | | |
| --- | --- | --- | --- | --- | --- | --- | --- | --- | --- | --- |
| Model |  | B | Standard error | Standardized coefficient - Beta | t | p | 95% C.I. Inf. Limit | 95% C.I. Sup. Limit | R^2^ | Durbin-Watson |
| 1 | Intercept | 1.313 | 0.170 |  | 7.735 | < 0.001 | 0.979 | 1.648 | 0.08 |  |
|  | SPQ-SF Vision | -0.025 | 0.020 | -0.118 | -1.246 | 0.214 | -0.064 | 0.014 |  |  |
|  | SPQ-SF Smell | 0.023 | 0.013 | 0.159 | 1.835 | 0.068 | -0.002 | 0.049 |  |  |
|  | SPQ-SF Taste | -0.031 | 0.024 | -0.105 | -1.288 | 0.199 | -0.079 | 0.016 |  |  |
|  | SPQ-SF Touch | -0.030 | 0.015 | -0.212 | -2.099 | 0.037 | -0.059 | -0.002 |  |  |
|  | SPQ-SF Hearing | 0.004 | 0.025 | 0.017 | 0.169 | 0.866 | -0.045 | 0.053 |  |  |
| 2 | Intercept | 1.108 | 0.545 |  | 2.034 | 0.043 | 0.035 | 2.182 | 0.123 | 2.262 |
|  | SPQ-SF Vision | -0.005 | 0.020 | -0.025 | -0.261 | 0.794 | -0.045 | 0.035 |  |  |
|  | SPQ-SF Smell | 0.015 | 0.013 | 0.101 | 1.175 | 0.241 | -0.010 | 0.040 |  |  |
|  | SPQ-SF Taste | -0.023 | 0.024 | -0.079 | -0.988 | 0.324 | -0.070 | 0.023 |  |  |
|  | SPQ-SF Touch | -0.026 | 0.014 | -0.183 | -1.826 | 0.069 | -0.055 | 0.002 |  |  |
|  | SPQ-SF Hearing | 0.020 | 0.025 | 0.077 | 0.784 | 0.434 | -0.030 | 0.069 |  |  |
|  | RAADS-R | 0.004 | 0.001 | 0.243 | 3.324 | 0.001 | 0.002 | 0.007 |  |  |
|  | Age | -0.024 | 0.025 | -0.058 | -0.961 | 0.338 | -0.072 | 0.025 |  |  |

| SWEAA Simultaneous Capacity | | | | | | | | | | |
| --- | --- | --- | --- | --- | --- | --- | --- | --- | --- | --- |
| Model |  | B | Standard error | Standardized coefficient - Beta | t | p | 95% C.I. Inf. Limit | 95% C.I. Sup. Limit | R^2^ | Durbin-Watson |
| 1 | Intercept | 1.183 | 0.208 |  | 5.677 | < 0.001 | 0.772 | 1.593 | 0.102 |  |
|  | SPQ-SF Vision | -0.065 | 0.024 | -0.247 | -2.652 | 0.009 | -0.112 | -0.017 |  |  |
|  | SPQ-SF Smell | 0.032 | 0.016 | 0.176 | 2.065 | 0.040 | 0.002 | 0.063 |  |  |
|  | SPQ-SF Taste | -0.002 | 0.030 | -0.006 | -0.070 | 0.944 | -0.060 | 0.056 |  |  |
|  | SPQ-SF Touch | 0.011 | 0.018 | 0.062 | 0.625 | 0.532 | -0.024 | 0.046 |  |  |
|  | SPQ-SF Hearing | -0.072 | 0.031 | -0.231 | -2.363 | 0.019 | -0.133 | -0.012 |  |  |
| 2 | Intercept | -0.613 | 0.644 |  | -0.953 | 0.342 | -1.881 | 0.655 | 0.207 | 1.958 |
|  | SPQ-SF Vision | -0.027 | 0.024 | -0.103 | -1.127 | 0.261 | -0.074 | 0.020 |  |  |
|  | SPQ-SF Smell | 0.017 | 0.015 | 0.095 | 1.154 | 0.250 | -0.012 | 0.047 |  |  |
|  | SPQ-SF Taste | 0.011 | 0.028 | 0.031 | 0.409 | 0.683 | -0.044 | 0.067 |  |  |
|  | SPQ-SF Touch | 0.023 | 0.017 | 0.129 | 1.355 | 0.176 | -0.010 | 0.056 |  |  |
|  | SPQ-SF Hearing | -0.050 | 0.029 | -0.161 | -1.708 | 0.089 | -0.108 | 0.008 |  |  |
|  | RAADS-R | 0.009 | 0.002 | 0.397 | 5.709 | < 0.001 | 0.006 | 0.012 |  |  |
|  | Age | 0.024 | 0.029 | 0.047 | 0.820 | 0.413 | -0.033 | 0.081 |  |  |

| SWEAA Pica | | | | | | | | | | |
| --- | --- | --- | --- | --- | --- | --- | --- | --- | --- | --- |
| Model |  | B | Standard error | Standardized coefficient - Beta | t | p | 95% C.I. Inf. Limit | 95% C.I. Sup. Limit | R^2^ | Durbin-Watson |
| 1 | Intercept | 0.405 | 0.100 |  | 4.031 | < 0.001 | 0.207 | 0.602 | 0.047 |  |
|  | SPQ-SF Vision | -0.023 | 0.012 | -0.188 | -1.960 | 0.051 | -0.046 | < 0.001 |  |  |
|  | SPQ-SF Smell | -0.009 | 0.008 | -0.108 | -1.227 | 0.221 | -0.024 | 0.006 |  |  |
|  | SPQ-SF Taste | -0.001 | 0.014 | -0.005 | -0.055 | 0.956 | -0.029 | 0.027 |  |  |
|  | SPQ-SF Touch | 0.003 | 0.009 | 0.038 | 0.365 | 0.716 | -0.014 | 0.020 |  |  |
|  | SPQ-SF Hearing | 0.006 | 0.015 | 0.038 | 0.382 | 0.703 | -0.023 | 0.035 |  |  |
| 2 | Intercept | 0.349 | 0.321 |  | 1.087 | 0.278 | -0.283 | 0.981 | 0.099 | 1.81 |
|  | SPQ-SF Vision | -0.011 | 0.012 | -0.088 | -0.904 | 0.367 | -0.034 | 0.013 |  |  |
|  | SPQ-SF Smell | -0.015 | 0.008 | -0.171 | -1.951 | 0.052 | -0.030 | < 0.001 |  |  |
|  | SPQ-SF Taste | 0.004 | 0.014 | 0.024 | 0.290 | 0.772 | -0.024 | 0.032 |  |  |
|  | SPQ-SF Touch | 0.006 | 0.008 | 0.067 | 0.662 | 0.508 | -0.011 | 0.022 |  |  |
|  | SPQ-SF Hearing | 0.016 | 0.015 | 0.107 | 1.066 | 0.287 | -0.013 | 0.045 |  |  |
|  | RAADS-R | 0.003 | 0.001 | 0.261 | 3.521 | 0.001 | 0.001 | 0.004 |  |  |
|  | Age | -0.018 | 0.014 | -0.077 | -1.276 | 0.203 | -0.047 | 0.010 |  |  |
